# Passivator-Free Microwave–Hydrothermal Synthesis of High Quantum Yield Carbon Dots for All-Carbon Fluorescent Nanocomposite Films

**DOI:** 10.3390/nano12152624

**Published:** 2022-07-29

**Authors:** Jiayin Wu, Qilin Lu, Hanchen Wang, Biao Huang

**Affiliations:** 1 College of Material Engineering, Fujian Agriculture and Forestry University, Fuzhou 350002, China; wujiayin@fafu.edu.cn (J.W.); wanghc08@163.com (H.W.); 2 Fujian Key Laboratory of Novel Functional Textile Fibers and Materials, Minjiang University, Fuzhou 350108, China

**Keywords:** chitosan, carbon dots, fluorescent composite film, nanocellulose, iron ion detection

## Abstract

Based on the self-passivation function of chitosan, an efficient, and green synthesis strategy was applied to prepare chitosan carbon dots (CDs). The quantum yield of carbon dots reached 35% under the conditions of hydrothermal temperature of 200 °C, hydrothermal time of 5 h, and chitosan concentration of 2%. Moreover, the obtained carbon dots had high selectivity and sensitivity to Fe^3+^. Based on the Schiff base reaction between the aldehyde groups of dialdehyde cellulose nanofibrils (DNF) and the amino groups of CDs, a chemically cross-linked, novel, fluorescent composite film, with high transparency and high strength, was created using one-pot processing. Knowing that the fluorescence effect of the composite film on Fe^3+^ had a linear relationship in the concentration range of 0–100 μM, a fluorescent probe can be developed for quantitative analysis and detection of Fe^3+^. Owing to their excellent fluorescent and mechanical properties, the fluorescent nanocomposite films have potential applications in the fields of Fe^3+^ detection, fluorescent labeling, and biosensing.

## 1. Introduction

Quantum dots (QDs), mainly II–VI semiconductor nanoparticles, have attracted extensive attention due to their unique optical properties [1], such as fluorescence tunability [2], bioimaging [3], photodynamic therapy [4] and photocatalysis [5]. However, their application in some fields, particularly biomedicine, is hindered by their their metal content, and the environmental impact of deposited QDs is also a concern. In contrast, carbon dots (CDs) are fluorescent carbon nanomaterials with a diameter of less than 10 nm, and are mainly composed of carbon nanoparticles [6,7]. CDs not only have the same excellent fluorescence performance as traditional metal-based quantum dots, but also have good biocompatibility and non-toxicity of carbon materials. Their remarkable properties of fluorescence tunability, water solubility, optical and physicochemical stability, give them potential for applications in chemical sensing [8,9], drug delivery [10,11], photocatalysis [12], bioimaging [13] and biosensing [14], and they are expected to replace traditional semiconductor quantum dots. Similar to semiconductor quantum dots, CDs have many electrons and holes on the surface, which endow the CDs with electron acceptor and electron donor roles to act as oxidizing or reducing agents [15,16]. In addition, benefiting from the numerous hydroxyl, amino or carboxyl groups on the surface, CDs can construct luminescent materials by coordinating or non-covalent bonding with high molecular polymers or inorganic compounds [17,18,19]. Relevant studies have shown that chitosan can be used as a passivator to improve the fluorescence performance of carbon dots [20]. Based on the self-passivating function of chitosan, CDs can be prepared by using chitosan as a carbon source without an additional passivating agent or further modification.

As functional materials, luminescent films have a wide range of applications in displays, light-emitting devices, safety signs and sensors. The fabrication of devices using QD nanoparticles usually requires their assembly in a matrix, and polymers are considered good candidates for host materials because they can improve dispersion, stability and processability, while retaining optical and electronic properties [21,22]. Among them, cellulose, as the most abundant natural polymer, has attracted much attention due to its unique mechanical properties, optical properties, economy, thermal stability, and expansion properties [23,24,25]. In this context, the research and development of quantum dots and cellulose nanocomposites [26], or nanocomposites with cellulose derivatives [27,28,29], is of special significance. Many membranes [30], hydrogels [31] and aerogels [32] with excellent fluorescence properties have been successfully prepared by using metal-based quantum dots as luminescent materials. However, the presence of metals, or their compounds, may lead to high cost and potential pollution, and it is essential to use carbon-based materials as an alternative for all-carbon luminescent composites. In recent years, luminescent films based on cellulose/carbon dots have become one of the research hotspots in this field. Junka et al. [33] demonstrated, for the first time, that CDs can be covalently bound to cellulose nanofibrils (CNFs) in aqueous media via EDC/NHS (N’-ethylcarbodiimide hydrochloride/*N*-hydroxysuccinimide) coupling. The surface modification of carboxymethylated-cellulose nanofibril films with CDs, by simple impregnation, resulted in transparent, smooth, and fluorescent nanopaper. The attachment of CDs endowed the films with higher thermal stability, lower roughness, and stronger fluorescence properties. By dispersing CDs in carboxymethyl cellulose (CMC) aqueous solution, You et al. [34] developed a transparent sunlight conversion film based on CMC and CDs, which has uniform structure, excellent optical properties and good tensile strength.

Herein, water-soluble chitosan carbon dots were synthesized greenly and efficiently by using a one-step microwave–hydrothermal method. Based on the self-passivation function of chitosan, the fluorescent carbon dots, with good fluorescence performance and high quantum yield, can be obtained without an additional passivation agent or further modification. The carbon dots were organically combined with dialdehyde cellulose nanofibrils (DNF) through the Schiff base reaction, to construct a novel fluorescent composite film with high transparency and high intensity. The resulting film has good fluorescence stability, a linear fluorescence response to Fe^3+^, and a high response selectivity and sensitivity, which can be used for Fe^3+^ quantitative analysis and detection.

## 2. Materials and Methods

### 2.1. Materials

Chitosan with deacetylation degree of 80~95% and viscosity of 50–800, Quinine sulfate, and ethylene glycol were supplied by Shanghai Aladdin Biochemical Technology Co., Ltd., Shanghai, China. Sodium periodate (NaIO_4_), sulfuric acid, acetic acid and FeCl_3_·7H_2_O were supplied by Sinopharm Chemical Reagent Co., Ltd., Shanghai, China. All reagents were analytical grade.

### 2.2. Preparation of DNF

**Figure 1:**
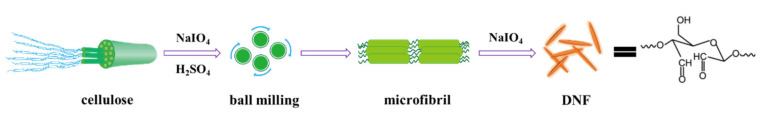
Schematic diagram of preparation of DNF.

A total of 4.0 g bamboo fibers were pretreated by ball milling at 500 rpm for 180 min in the presence of sulfuric acid solution at pH 3.56 and 2.0 g NaIO_4_. Then, with the addition of 4.0 g NaIO_4_, the reaction was continued at 350 rpm for 60 min to fully oxidize cellulose. After the ball-milling, the sample was washed out with deionized water, mixed with 25 mL ethylene glycol, and magnetically stirred in the dark for 120 min to remove unreacted NaIO_4_. The samples were repeatedly centrifuged and washed with deionized water at 9000 rpm until the supernatant was neutral. The upper milky white suspension, namely DNF, was collected by centrifugation at 5000 rpm. The preparation mechanism is shown in Figure 1. According to Lindh’s [35] method, the content of DNF aldehyde group was determined to be 5.326 mmol/g, corresponding to an oxidation degree of 42.6%.

### 2.3. Chitosan Carbon Dots (CDs) Synthesis

**Figure 2:**
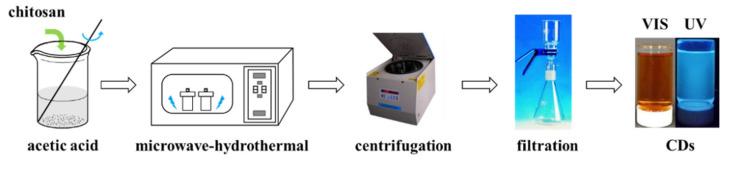
Schematic diagram of synthesis of CDs solution.

A total of 2 g chitosan was fully dissolved in 100 mL 1% (*w*/*w*) acetic acid solution, and the mixture was sealed in a high-temperature and high-pressure reactor under argon protection. The high-pressure reactor was placed in a microwave synthesis reactor for 5 h, with microwave power of 500 W and reaction temperature of 220 °C. A large amount of deionized water was then added to the reacted mixture and centrifuged at 10,000 rpm for 30 min to separate the yellow-brown product. The supernatant was successively filtered through 0.45 μm and 0.2 μm filters to remove impurities, and the remaining filtrate was chitosan carbon dots solution (Figure 2). The quantum yield (*Q*) of chitosan carbon dots was calculated using the method of reference [36]. Quinine sulfate dissolved in 0.1 M H_2_SO_4_ solution (*Q*_s_ = 0.54) was used as the standard, and the fluorescence emission spectrum and ultraviolet absorption spectrum of the reference substance and CDs were scanned. The relative quantum yield of CDs can be calculated with the following equation:

(1) $$Q_{x}=Q_{s}\times \frac{F_{x}}{F_{s}}\times \frac{A_{s}}{A_{x}}$$

where *Q* is the quantum yield, *F* represents the peak area of the emission spectrum when the excitation wavelength Ex = 335 nm, and *A* is the absorbance of the sample at the excited wavelength of 335 nm. The subscripted “*s*” and “*x*” refer to the reference substance and the test samples, respectively.

### 2.4. Construction of All-Carbon Fluorescent Nanocomposite Films

**Figure 3:**
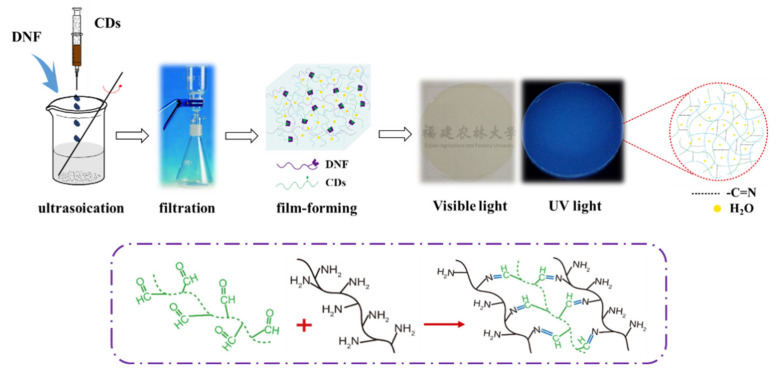
Schematic diagram of preparation of fluorescent composite film.

A measured quantity of CDs and DNF suspensions (solid content 1.12%) were mixed and stirred in an ultrasonic reactor at 50 °C for 60 min. The mixture was suction filtered through a 0.1 μm filter membrane into a film with a thickness of 0.2 mm. The formed film was dried in a constant temperature and humidity oven at 50 °C for 24 h to obtain an all-carbon fluorescent composite film. The schematic diagram of the preparation process is shown in Figure 3. For the control experiments, fluorescent nanocomposite films with different CD contents, but the same amount of DNF, were prepared and designated as F-*n*, where *n* indicates the percentage of m_CDs_/m_DNF_.

### 2.5. Characterization

The absorption spectra and transmittance of CDs solution and composite films were recorded on a UV-3200 UV-vis spectrophotometer (Mapada Instruments Co., Shanghai, China). The scanning wavelength range was 200–600 nm and the slit width was set at 2 nm. The optimal excitation wavelength and optimal emission wavelength of CDs solution were obtained by using an F-7000 fluorescence spectrometer (Hitachi, Ltd., Tokyo, Japan) with a scanning rate of 240 nm/min and a scanning frequency of 40 Hz at slits of 5/5 nm. The fluorescence properties of the composite film were characterized by setting excitation wavelength to 335 nm and emission wavelength range of 350–650 nm. An appropriate amount of FeCl_3_·7H_2_O was dissolved in deionized water to prepare Fe^3+^ solutions with different concentrations, and the responsiveness of CDs and composite membranes to iron ions was investigated by the change in fluorescence intensity after adding Fe^3+^ solution.

After diluting DNF and CDs solution, the microstructure of DNF and CDs was observed on a Tecnai G2-F20 Transmission Electron Microscope (FEI Co., Hillsboro, OR, USA) and a NanoScope IIIa MultiMode Atomic Force Microscope (Veeco Co., Plainview, NY, USA). After freeze-drying, the surface and cross section morphologies of the composite film were characterized using a Nova Nano SEM 230 (FEI Co.) and AFM.

The surface functional groups of the fluorescent composite films were characterized using a Nicolet 380 Fourier Transform Infrared spectroscopy (FTIR) (Thermo electron Instruments Co., Ltd., Waltham, MA, USA), in the form of KBr pellets with a resolution of 4 cm^−1^. Thermal stability was analyzed in a thermogravimetric analyzer (TGA) (NETZSCH STA 449 F3 Jupiter^®^, Selb, Germany), heating from 30 °C to 700 °C at a rate of 10 °C/min in a N_2_ atmosphere of 30 mL/min. The zeta potential of DNF and CDs was measured using an SZP-06 Zeta potential analyzer (Mütek, Filderstadt, Germany), and the positive and negative charges of DNF and CDs were analyzed by detecting the zeta potential.

## 3. Results and Discussion

### 3.1. Fluorescence Properties of CDs

**Figure 4:**
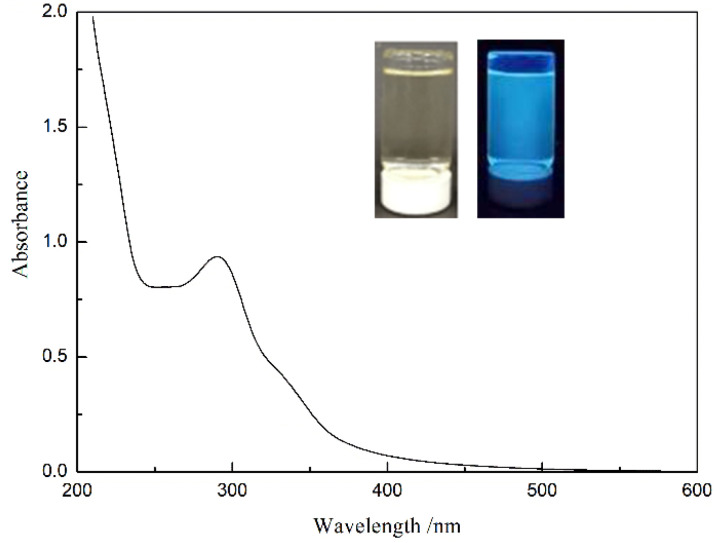
UV absorption spectrum of CDs solution.

**Figure 5:**
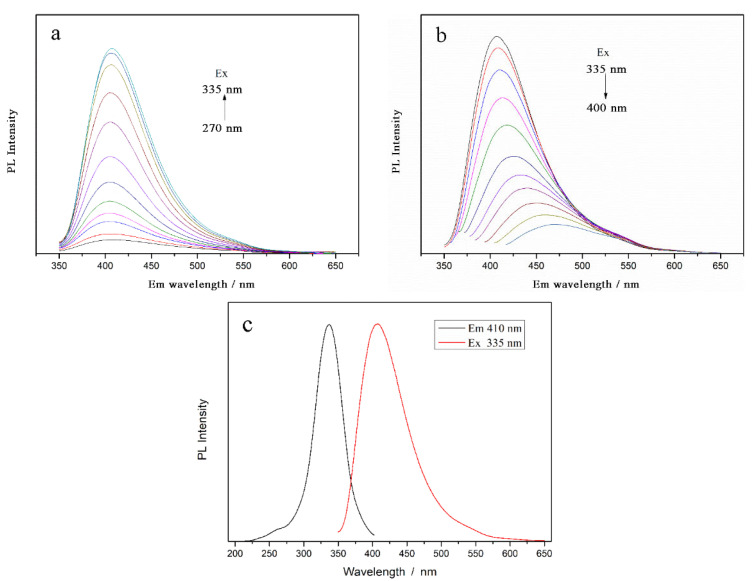
Emission spectra of CDs at different excitation wavelengths of (**a**) 270–335 nm and (**b**) 335–400 nm; (**c**) Emission spectra and excitation spectra of CDs (Ex = 335 nm, Em = 410 nm).

As shown in Figure 4, the diluted CDs solution is transparent and clear pale yellow under natural light, and shows an excellent fluorescence effect, with strong blue fluorescence under UV excitation. The CDs have a strong UV-vis absorption feature centered at 292 nm, assigned to the n→π* transition of the surface moieties [37,38]. The phenomenon of emission spectra changing with the excitation wavelength (Figure 5a,b) is similar to that reported by Sun et al. [39]. Surface passivation may generate defect sites on the surface of carbon nanoparticles, and the defect sites can capture radiative excitons, causing fluorescence emissions [16,40,41]. The formation and surface functionalization of carbon nanoparticles occur simultaneously during hydrothermal carbonization. Abundant functional groups, such as carboxylic acids and amino groups, can introduce various defects on the surface, acting as excitation energy holes to induce a fluorescence effect. The results show that the best excitation and emission wavelengths corresponding to the maximum fluorescence intensity are 335 nm and 410 nm, respectively. Interestingly, when the excitation wavelength is higher than 335 nm, the fluorescence emission intensity, at about 530 nm, becomes relatively obvious with the increase in excitation wavelength and the decrease in peak fluorescence intensity, which cannot be ignored at excitation wavelengths over 400 nm. Emission spectra of the CDs solution at the optimal excitation wavelength (335 nm), and excitation spectra at the optimal excitation wavelength (410 nm), are shown in Figure 5c. It can be seen that there is a large energy difference between the excitation peak and the emission peak, and the quantum yield of CDs is calculated to be about 35%.

### 3.2. Morphology of DNF and CDs

**Figure 6:**
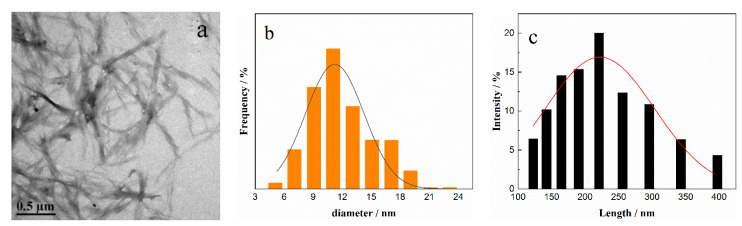
(**a**) TEM image of DNF; distribution of (**b**) diameter and (**c**) length of DNF.

**Figure 7:**
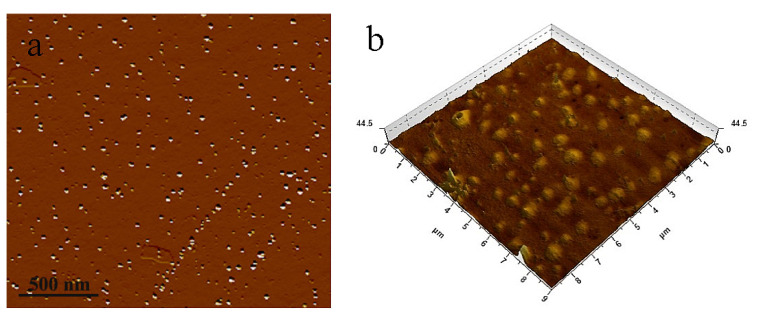
AFM images of CDs (**a**) 2D image and (**b**) 3D image (scan area 2 × 2 μm^2^).

DNF is rod-shaped, with a length of 100–300 nm and a diameter of 5–15 nm, and the average values are 220 nm and 11 nm, respectively (Figure 6). Figure 7 shows the 2D and 3D images of CDs. CDs are spherical nanoparticles with diameters of 3–10 nm. The Zeta potentials of DNF and CDs were −32.6 mV and 77.6 mV, respectively. The higher the absolute value of Zeta potential, the stronger the electrostatic repulsion, which is conducive to better particle dispersion in water and to lower agglomeration. The higher Zeta potential of CDs is due to the presence of amino groups on the surface, indicating that the hydrothermal carbonization of chitosan is an effective method to synthesize amino functionalized fluorescent carbon dots.

### 3.3. Iron Ion Responsiveness of CDs

**Figure 8:**
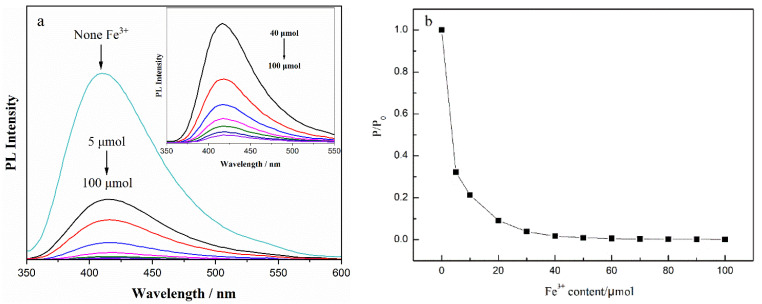
(**a**) Emission spectra and (**b**) PL intensity change in CDs with different Fe^3+^ content (Ex = 335 nm); Inset: emission spectra of CDs with Fe^3+^ content ranging from 40 to 100 μmol.

As shown in Figure 8a, the fluorescence-quenching effect of Fe^3+^ on CDs was analyzed using the emission spectra of CDs solution at Ex = 335 nm, with the addition of 1 mL Fe^3+^ solution of different concentrations (0–100 μmol/mL), to 1 mL CDs solution. According to the calculation of the ratio P/P_0_ of the fluorescence intensity of the CDs solution with different Fe^3+^ additions to that without Fe^3+^ ions, the fluorescence-quenching of the CDs was analyzed, as shown in Figure 8b. With a trace amount of Fe^3+^ ions (5 μmol), the fluorescence intensity of the CDs solution decreased to 30% of the original solution. When Fe^3+^ content was more than 30 μmol, the fluorescence effect of the CDs solution almost disappeared, indicating that the obtained CDs have sensitivity and high selectivity to Fe^3+^ ions.

### 3.4. Mechanical Properties of Composite Films

**Figure 9:**
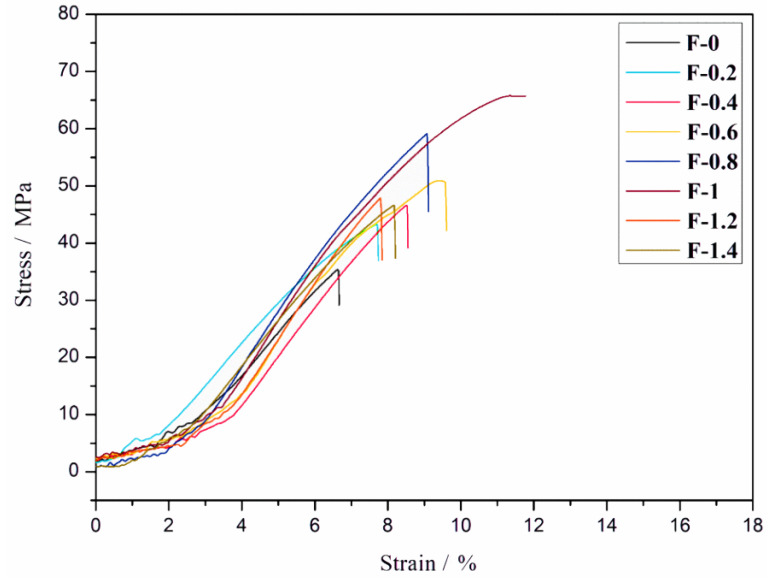
Stress and strain curves of composite films with different amounts of CDs.

It can be seen from Figure 9 that the tensile stress and strain of the composite film first increases and then decreases with the increase in the addition of CDs. When m_CDs_/m_DNF_ was 1%, the tensile stress of the fluorescent composite film reached the maximum of 70.4 MPa, which was two times higher than that without CDs (35.1 MPa), and the strain was 13.1%. This is mainly because, with the increase in CDs’ dosage, the chemical crosslinking degree of DNF and CDs derived from the Schiff base bond increases, and the interaction between them is enhanced, thereby significantly improving the tensile strength of the composite films. When m_CDs_/m_DNF_ was higher than 1%, excessive CDs led to an increase in the probability of collision between them, which caused some CDs to agglomerate through hydrogen bonds, thus reducing the mechanical properties of composite films. Therefore, an appropriate quantity of CDs can effectively improve the mechanical strength of the fluorescent composite films.

### 3.5. Optical Performance Analysis of Composite Films

**Figure 10:**
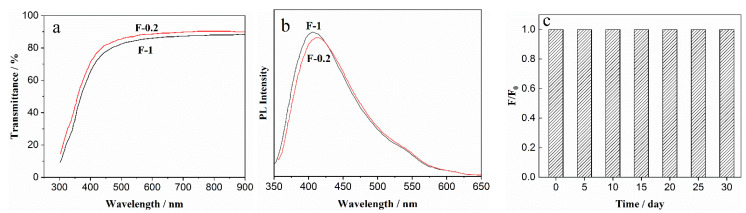
(**a**) Light transmittance curves of the composite films F-0.2 and F-1 (**b**) Emission spectra of F-0.2 and F-1; (**c**) PL intensity changes with time increase in F-1 (Ex = 335 nm).

As shown in Figure 10a, compared with the F-0.2 film, the light transmittance of the F-1 composite film with CDs declines in the visible light region. This may be because, as the content of CDs increases, more Schiff base bonds are formed between CDs and DNF, and the internal structure of the composite film is more compact. The reduced transmittance of F-1 film is still higher than 85%, indicating that the composite film can maintain high transparency in the presence of CDs, and it therefore has application potential as a transparent luminous film. Figure 10b shows the emission spectra of composite films F-0.2 and F-1 at Ex = 335 nm. With increasing CDs content, the fluorescence intensity of the composite film increases slightly, which enhances the composite film as a luminescent film. The changing trend of the fluorescence intensity of the composite film F-1 over time (placed at room temperature without UV light), is shown in Figure 10c. The fluorescence intensity of F-1 film had almost no change one month later, indicating that the composite film has excellent fluorescence stability.

### 3.6. Morphology of Composite Films

**Figure 11:**
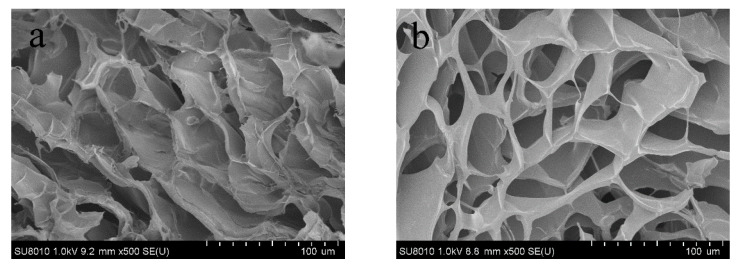
Cross-section SEM images of composite film (**a**) F-0 and (**b**) F-1.

**Figure 12:**
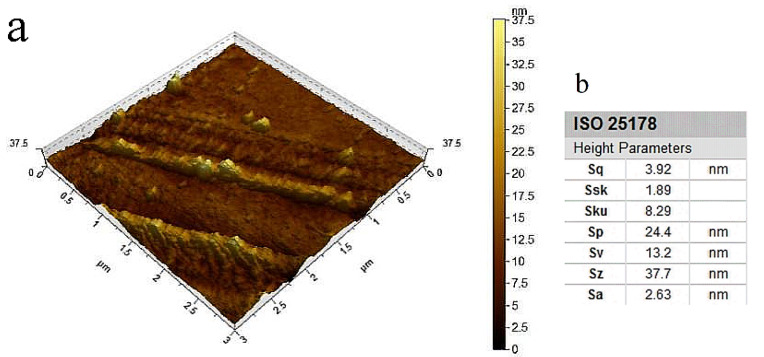
(**a**) AFM 3D image and (**b**) surface roughness parameter table of the composite film F-1.

As shown in Figure 11, F-1 film displayed a more compact structure than F-0 film, contributing to the stronger mechanical properties of F-1 film. The Schiff base bond between CDs and DNF increases the internal network density of the composite film, and also creates more intermolecular forces to form more cross-linking sites, which, in turn, affect the pore structure, pore size and distribution of the film [42]. The AFM image and surface roughness parameter of F-1 film are shown in Figure 12. The surface of F-1 film was smooth, with the root square roughness (Sq) of 3.92 nm [34,43]. The surface kurtosis (Sku) of the composite film exceeded 3, implying that the surface morphology distribution is concentrated and the surface structure was relatively uniform [44]. DNF and CDs are well cross-linked in the F-1 film and are evenly distributed without agglomeration to show good compatibility.

### 3.7. FTIR

**Figure 13:**
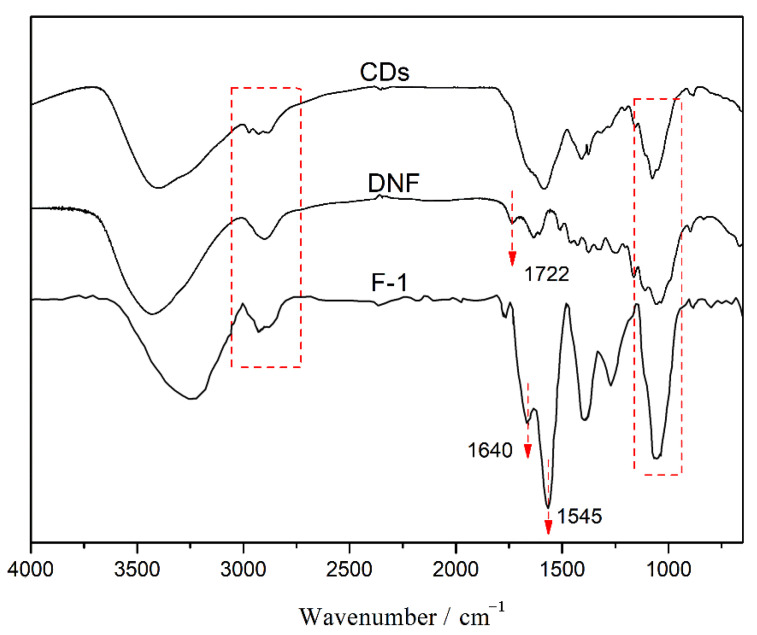
FTIR spectra of CD, DNF and the composite film F-1.

In order to further explore the formation mechanism of the internal network of the composite film, the infrared spectra are shown in Figure 13, which shows that F-1 composite film has the characteristic absorption peaks of both DNF and CDs. The absorption peak at 1722 cm^−1^ is attributed to the stretching vibration of C=O of DNF and the peaks around 1060 cm^−1^ belong to the C–O stretching vibration of DNF and CDs. The peaks at 2800–2900 cm^−1^ can be assigned to the stretching vibration of C–H. The peaks near 3404 cm^−1^ correspond to the stretching vibration of O–H and N–H, while that of F-1 exhibits a redshift, which may be caused by the formation of Schiff base bonds. The strong absorption peak at 1545 cm^−1^ in F-1 film spectrum is attributed to the stretching vibration of the Schiff base bond (C=N) [45], which confirms the covalent bonding of DNF and CDs in the composite film, and is beneficial in improving the mechanical properties of the fluorescent composite film.

### 3.8. TGA

**Figure 14:**
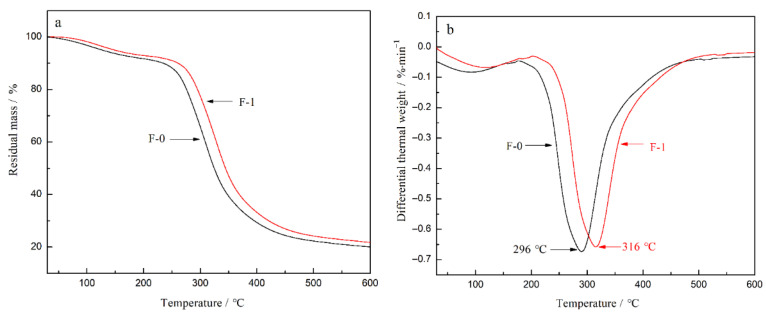
(**a**) TG curves and (**b**) DTG curves of the composite films F-0 and F-1.

Figure 14 shows the thermogravimetry (TG) and derivative thermogravimetry (DTG) curves of F-0 film and F-1 film. For F-1 film, the onset temperature of thermal decomposition is 262 °C, the temperature at the maximum weight loss rate is 316 °C, and the residual mass is 22.1%. the corresponding values of the F-0 film are lower than those of F-1 film, being 238 °C, 296 °C and 20.3%, respectively. This phenomenon illustrates the fact that the thermal stability of the F-1 film is enhanced compared to that of F-0 film. This is due to the chemical cross-linking and hydrogen bonding between CDs and DNF, which is conducive to a denser internal structure and better thermal stability of the fluorescent composite film.

### 3.9. Responsiveness of Fluorescent Composite Film to Fe^3+^

**Figure 15:**
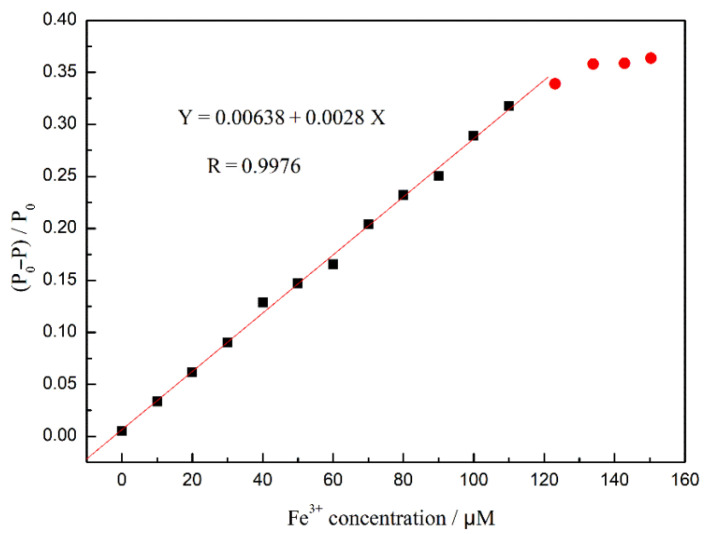
A linear regression curve of the films F-1 in response to Fe^3+^ concentration (Ex = 350 nm).

Based on the interaction between Fe^3+^ and fluorescent CDs, the sensitivity of the F-1 film to Fe^3+^ was investigated. An Fe^3+^ solution (0–150 μM) was prepared to observe the effect of different concentrations of Fe^3+^ ions on the fluorescence intensity of the composite film. According to the fluorescence-quenching degree, the change in the fluorescence intensity of the composite films with different concentrations of Fe^3+^ ions was linearly fitted, and the regression curve is shown in Figure 15. The fluorescence quenching efficiency of F-1 film shows a linear response to Fe^3+^ ions in the concentration range of 0–100 μM, indicating that the fluorescence intensity of the composite film can be effectively quenched by adding Fe^3+^ ions. The calibration curve can be expressed as Y = 0.00638 + 0.0028 X. The limit of detection (LOD) of Fe^3+^ is calculated to be 9.09 nM, using the 3σ criterion, which is lower than the detection limits of most other research [46,47,48,49,50]. Therefore, the fluorescence composite film has a high sensitivity to Fe^3+^ and can be applied to detect trace Fe^3+^ in the field of sensing and environmental monitoring.

## 4. Conclusions

Based on the self-passivation function of chitosan, chitosan CDs with good fluorescent properties were synthesized using the microwave–hydrothermal method without passivators and strong acid solvents. This strategy is low-cost and environmentally friendly, which provides a new method for the large-scale preparation of highly aminated fluorescent CDs. The quantum yield of CDs is up to 35%, and their fluorescence quenching effect on Fe^3+^ is sensitive. A fluorescent composite film with high strength and high transparency was constructed based upon the Schiff base reaction between CDs and DNF. The existence of CDs can improve the mechanical properties and thermal stability of the composite film. Owing to the sensitive linear response to Fe^3+^ concentration, the composite film can be used in the quantitative analysis and detection of Fe^3+^ in environmental monitoring and biosensing fields.

## Data Availability

The data presented in this study are available on request from the corresponding author.

## References

[1] Ledentsov N.N., Ustinov V.M., Shchukin V.A., Kop’ev P.S., Alferov Z.I., Bimberg D. (1998). Quantum dot heterostructures: Fabrication, properties, lasers. Semiconductors.

[2] Padilha L.A., Stewart J.T., Sandberg R.L., Bae W.K., Koh W.K., Pietryga J.M., Klimov V.I. (2013). Carrier multiplication in semiconductor nanocrystals: Influence of size, shape, and composition. Acc. Chem. Res..

[3] Biju V. (2014). Chemical modifications and bioconjugate reactions of nanomaterials for sensing, imaging, drug delivery and therapy. Chem. Soc. Rev..

[4] Jia X., JIA L. (2012). Nanoparticles improve biological functions of phthalocyanine photosensitizers used for photodynamic therapy. Curr. Drug Metab..

[5] Tada H., Fujishima M., Kobayashi H. (2011). Photodeposition of metal sulfide quantum dots on titanium(IV) dioxide and the applications to solar energy conversion. Chem. Soc. Rev..

[6] Hu Q., Paau M.C., Zhang Y., Chan W., Gong X., Zhang L., Choi M.M. (2013). Capillary electrophoretic study of amine/carboxylic acid-functionalized carbon nanodots. J. Chromatogr. A.

[7] Tuerhong M., Xu Y., Yin X.B. (2017). Review on carbon dots and their applications. Chin. J. Anal. Chem..

[8] Qian Z., Shan X., Chai L., Ma J., Chen J., Feng H. (2014). Si-doped carbon quantum dots: A facile and general preparation strategy, bioimaging application, and multifunctional sensor. ACS Appl. Mater. Interfaces.

[9] Zhang L., Han Y., Zhu J., Zhai Y., Dong S. (2015). Simple and sensitive fluorescent and electrochemical trinitrotoluene sensors based on aqueous carbon dots. Anal. Chem..

[10] Ding H., Du F., Liu P., Chen Z., Shen J. (2015). DNA–carbon dots function as fluorescent vehicles for drug delivery. ACS Appl. Mater. Interfaces.

[11] Chen P., Wang Z., Zong S., Zhu D., Chen H., Zhang Y., Wu L., Cui Y. (2016). pH-sensitive nanocarrier based on gold/silver core-shell nanoparticles decorated multi-walled carbon manotubes for tracing drug release in living cells. Biosens. Bioelectron..

[12] Chang C.M., Orchard K.L., Martindale B.C., Reisner E. (2016). Ligand removal from CdS quantum dots for enhanced photocatalytic H_2_ generation in pH neutral water. J. Mater. Chem. A.

[13] Xu J.J., Zhao W.W., Song S., Fan C., Chen H.Y. (2014). Functional nanoprobes for ultrasensitive detection of biomolecules: An update. Chem. Soc. Rev..

[14] Lin L., Rong M., Luo F., Chen D., Wang Y., Chen X. (2014). Luminescent graphene quantum dots as new fluorescent materials for environmental and biological applications. TrAC Trends Anal. Chem..

[15] Tian L., Ghosh D., Chen W., Pradhan S., Chang X., Chen S. (2009). Nanosized carbon particles from natural gas soot. Chem. Mater..

[16] Han S., Zhang H., Xie Y., Liu L., Shan C., Li X., Liu W., Tang Y. (2015). Application of cow milk-derived carbon dots/Ag NPs composite as the antibacterial agent. Appl. Surf. Sci..

[17] Shen L., Chen M., Hu L., Chen X., Wang J. (2013). Growth and stabilization of silver nanoparticles on carbon dots and sensing application. Langmuir.

[18] Zairov R.R., Dovzhenko A.P., Sarkanich K.A., Nizameev I.R., Luzhetskiy A.V., Sudakova S.N., Podyachev S.N., Burilov V.A., Vatsouro I.M., Vomiero A. (2021). Single excited dual band luminescent hybrid carbon dots-terbium chelate nanothermometer. Nanomaterials.

[19] Bochkova O., Dovjenko A., Zairov R., Kholin K., Biktimirova R., Fedorenko S., Nizameev I., Laskin A., Voloshina A., Lyubina A. (2022). Silica-supported assemblage of Cu^II^ ions with carbon dots for self-boosting and glutathione-induced ROS generation. Coatings.

[20] Tan M., Zhang L., Tang R., Song X., Li Y., Wu H., Wang Y., Lv G., Liu X., Ma X. (2013). Enhanced photoluminescence and characterization of multicolor carbon dots using plant soot as a carbon source. Talanta.

[21] Ostermann J., Merkl J.P., Flessau S., Wolter C., Kornowksi A., Schmidtke C., Pietsch A., Kloust H., Feld A., Weller H. (2013). Controlling the physical and biological properties of highly fluorescent aqueous quantum dots using block copolymers of different size and shape. ACS Nano.

[22] Qian L., Zheng Y., Xue J., Holloway P.H. (2011). Stable and efficient quantum-dot light-emitting diodes based on solution-processed multilayer structures. Nat. Photonics.

[23] Dufresne A. (2013). Nanocellulose: A new ageless bionanomaterial. Mater. Today.

[24] Lu Q., Tang L., Lin F., Wang S., Chen Y., Chen X., Huang B. (2014). Preparation and characterization of cellulose nanocrystals via ultrasonication-assisted FeCl_3_-catalyzed hydrolysis. Cellulose.

[25] Siró I., Plackett D. (2010). Microfibrillated cellulose and new nanocomposite materials: A review. Cellulose.

[26] Yang Z., Chen S., Hu W., Yin N., Zhang W., Xiang C., Wang H. (2012). Flexible luminescent CdSe/bacterial cellulose nanocomoposite membranes. Carbohydr. Polym..

[27] Abitbol T., Gray D.G. (2009). Incorporation into paper of cellulose triacetate films containing semiconductor nanoparticles. Cellulose.

[28] Abitbol T., Wilson J.T., Gray D.G. (2011). Electrospinning of fluorescent fibers from CdSe/ZnS quantum dots in cellulose triacetate. J. Appl. Polym. Sci..

[29] Abitbol T., Gray D. (2007). CdSe/ZnS QDs Embedded in Cellulose Triacetate Films with Hydrophilic Surfaces. Chem. Mater..

[30] Luna-Martínez J.F., Hernández-Uresti D.B., Reyes-Melo M.E., Guerrero-Salazar C.A., González-González V.A., Sepúlveda-Guzmán S. (2011). Synthesis and optical characterization of ZnS–sodium carboxymethyl cellulose nanocomposite films. Carbohydr. Polym..

[31] Dou H., Yang W., Tao K., Li W., Sun K. (2010). Thermal sensitive microgels with stable and reversible photoluminescence based on covalently bonded quantum dots. Langmuir.

[32] Wang H., Shao Z., Bacher M., Liebner F., Rosenau T. (2013). Fluorescent cellulose aerogels containing covalently immobilized (ZnS)_x_(CuInS_2_)_1−x_/ZnS (core/shell) quantum dots. Cellulose.

[33] Junka K., Guo J., Filpponen I., Laine J., Rojas O.J. (2014). Modification of cellulose nanofibrils with luminescent carbon dots. Biomacromolecules.

[34] You Y., Zhang H., Liu Y., Lei B. (2016). Transparent sunlight conversion film based on carboxymethyl cellulose and carbon dots. Carbohydr. Polym..

[35] Lindh J., Carlsson D.O., Strømme M., Mihranyan A. (2014). Convenient one-pot formation of 2,3-dialdehyde cellulose beads via periodate oxidation of cellulose in water. Biomacromolecules.

[36] Wang C., Lin H., Xu Z., Huang Y., Humphrey M.G., Zhang C. (2016). Tunable carbon-dot-based dual-emission fluorescent nanohybrids for ratiometric optical thermometry in living cells. ACS Appl. Mater. Interfaces.

[37] Yang Y., Cui J., Zheng M., Hu C., Tan S., Xiao Y., Liu Y. (2012). One-step synthesis of amino-functionalized fluorescent carbon nanoparticles by hydrothermal carbonization of chitosan. Chem. Commun..

[38] Sonsin A.F., Nascimento S.M.S., Albuquerque I.M.B., Silva E.C.O., Rocha J.C.A., Oliveira R.S., Barbosa C.A.E.S., Souza S.T., Fonseca E.J.S. (2021). Temperature-dependence on the optical properties of chitosan carbon dots in the solid state. RSC Adv..

[39] Sun Y.P., Zhou B., Lin Y., Wang W., Fernando K.A.S., Pathak P., Meziani K.J., Harruff B.A., Wang X., Wang H. (2006). Quantum-sized carbon dots for bright and colorful photoluminescence. J. Am. Chem. Soc..

[40] Yang S.T., Cao L., Luo P.G., Lu F., Wang X., Wang H., Meziani M.J., Liu Y., Qi G., Sun Y.P. (2009). Carbon dots for optical imaging in vivo. J. Am. Chem. Soc..

[41] Ray S.C., Saha A., Jana N.R., Sarkar R. (2009). Fluorescent carbon nanoparticles: Synthesis, characterization, and bioimaging application. J. Phys. Chem. C.

[42] Nieto-Suárez M., López-Quintela M.A., Lazzari M. (2016). Preparation and characterization of crosslinked chitosan/gelatin scaffolds by ice segregation induced self-assembly. Carbohydr. Polym..

[43] Jing Z., Yan L. (2015). Metal-free transparent luminescent cellulose films. Cellulose.

[44] Sharifi-Viand A., Mahjani M.G., Jafarian M. (2014). Determination of fractal rough surface of polypyrrole film: AFM and electrochemical analysis. Synth. Met..

[45] Lu T., Li Q., Chen W., Yu H. (2014). Composite aerogels based on dialdehyde nanocellulose and collagen for potential applications as wound dressing and tissue engineering scaffold. Compos. Sci. Technol..

[46] Zhang Q., Sun Y., Liu M., Liu Y. (2020). Selective detection of Fe^3+^ ions based on fluorescence MXene quantum dots via a mechanism integrating electron transfer and inner filter effect. Nanoscale.

[47] Chen M., An J., Hu Y., Chen R., Lyu Y., Hu N., Luo M., Yuan M., Liu Y. (2020). Swelling-shrinking modified hyperstatic hydrophilic perovskite polymer fluorescent beads for Fe (III) detection. Sens. Actuators B.

[48] Guo X., Pan Q., Song X., Guo Q., Zhou S., Qiu J., Dong G. (2021). Embedding carbon dots in Eu^3+^-doped metal-organic framework for label-free ratiometric fluorescence detection of Fe^3+^ ions. J. Am. Ceram. Soc..

[49] Chen Z., Xu X., Meng D., Jiang H., Zhou Y., Feng S., Mu Z., Yang Y. (2020). Dual-emitting N/S-doped carbon dots-based ratiometric fluorescent and light scattering sensor for high precision detection of Fe (III) ions. J. Fluoresc..

[50] Mohajer F., Ziarani G.M., Badiei A., Ghasemi J.B., Varma R.S., Karimi-Maleh H. (2022). Pomegranate Punica granatum peel waste as a naked-eye natural colorimetric sensor for the detection and determination of Fe^3+^ and I^−^ ions in water. Chemosphere.

